# Therapeutic vaccines for herpesviruses

**DOI:** 10.1172/JCI179483

**Published:** 2024-05-01

**Authors:** Jeffrey I. Cohen

**Affiliations:** Laboratory of Infectious Diseases, National Institute of Allergy and Infectious Diseases, NIH, Bethesda, Maryland, USA.

## Abstract

Herpesviruses establish latent infections, and most reactivate frequently, resulting in symptoms and virus shedding in healthy individuals. In immunocompromised patients, reactivating virus can cause severe disease. Persistent EBV has been associated with several malignancies in both immunocompromised and nonimmunocompromised persons. Reactivation and shedding occur with most herpesviruses, despite potent virus-specific antibodies and T cell immunity as measured in the blood. The licensure of therapeutic vaccines to reduce zoster indicates that effective therapeutic vaccines for other herpesviruses should be feasible. However, varicella-zoster virus is different from other human herpesviruses in that it is generally only shed during varicella and zoster. Unlike prophylactic vaccines, in which the correlate of immunity is antibody function, T cell immunity is the correlate of immunity for the only effective therapeutic herpesvirus vaccine–zoster vaccine. While most studies of therapeutic vaccines have measured immunity in the blood, cellular immunity at the site of reactivation is likely critical for an effective therapeutic vaccine for certain viruses. This Review summarizes the status of therapeutic vaccines for herpes simplex virus, cytomegalovirus, and Epstein-Barr virus and proposes approaches for future development.

## Introduction

Herpesvirus infections persist for the lifetime of the individual and may be associated with significant morbidity when they reactivate or are associated with malignancies (Table 1). Reactivation of herpes simplex virus (HSV) results in recurrent genital or orolabial HSV and visceral disease in immunocompromised patients. Reactivation of varicella-zoster virus (VZV) results in zoster and organ disease in immunocompromised patients. Reactivation of cytomegalovirus (CMV) can cause severe organ disease in transplant recipients. Epstein-Barr virus (EBV) is associated with several epithelial cell carcinomas and B cell lymphomas; in these cancers at least one viral gene is expressed in each of the tumor cells. Numerous clinical trials of therapeutic vaccines to prevent reactivation have been performed for HSV, CMV, and EBV, but none have been licensed.

Therapeutic herpesvirus vaccines might have additional benefits beyond preventing virus reactivation or, in the case of EBV, treating virus-associated malignancies. Chronic virus infections may perturb the immune system, resulting in increased risks for autoimmune disease. EBV is a risk factor for multiple sclerosis (1). Chronic virus infections have also been linked to persistent inflammation; several studies reported an increased risk of dementia in patients with chronic herpesvirus infections (2). Persons receiving a live-attenuated zoster vaccine virus have been reported to have a lower risk of dementia (3). This Review evaluates the history of therapeutic vaccines for human herpesviruses and proposes principles for vaccine development.

## Proof of principle: feasibility of therapeutic herpesvirus vaccines

The success of two therapeutic vaccines for zoster proves that a therapeutic vaccine is possible for herpesviruses. While antibody function is the correlate of protection for licensed viral vaccines that protect against primary infection, including the varicella vaccine (4), VZV-specific CD4 cells are thought to be the mechanistic correlate of protection for VZV (5). Both the live-attenuated zoster vaccine (Zostavax) and subunit zoster vaccine (VZV glycoprotein E formulated with AS01b adjuvant, Shingrix) reduce the incidence of zoster (6). These two vaccines induce virus-specific CD4^+^ and CD8^+^ T cell responses to VZV (7) and antibody that mediates antibody-dependent cellular cytotoxicity (ADCC) (8), both of which are important for killing virus-infected cells. In addition, VZV glycoprotein E functions as a viral Fc receptor and antibodies against glycoprotein E may block viral Fc receptor activity, resulting in enhanced ADCC.

Therapeutic vaccines have not been licensed for HSV, CMV, or EBV. However, the success of infusion of HLA-matched, virus-specific T cells in reducing viremia and treating disease associated with reactivation of CMV and EBV (posoleucel, ref. 9) indicates that if a vaccine can induce similar virus-specific T cell responses then it should be effective.

Several qualities of virus-specific T cells are felt to be important for their activity, and presumably these features would be important for therapeutic vaccines (10). First, both CD4^+^ and CD8^+^ effector T cells should be present to ensure optimal antiviral activity. Second, more than one viral epitope should be recognized to reduce the risk of impaired effectiveness owing to viral antigen mutants or polymorphisms. Third, virus-specific T cells must recognize viral antigens presented in association with both class I and II MHC antigens. Based on the success of virus-specific T cells in reducing disease associated with CMV and EBV, induction of these cells by a vaccine should be an important goal for therapeutic vaccines.

## Difficulties for HSV, CMV, and EBV therapeutic vaccines

The effectiveness of two vaccines in preventing shingles suggests that therapeutic vaccines for other herpesviruses may be possible. However, VZV has a number of differences with other human herpesviruses for which therapeutic vaccines have not yet been approved. Unlike other human herpesviruses, VZV is shed and transmitted much less frequently from virus-infected persons who do not have symptomatic disease (varicella or zoster). In the absence of vaccination, about 50% of persons aged 80 or older will have one episode of zoster in their lifetime. Thus, in older unvaccinated individuals without reexposure to VZV, the immune system may not have been exposed to VZV in more than 50 years after primary infection. Therefore, a therapeutic vaccine for zoster may induce a large increase in VZV-specific immunity in adults.

HSV, CMV, and EBV are shed in saliva very frequently, and, therefore, the immune system is often exposed to replicating virus. In a study of patients with predominantly asymptomatic HSV who swabbed their oral and genital mucosa four times daily, 20.5% of genital swabs and 11.6% of oral swabs were positive for HSV (11). Thus, despite HSV frequently reactivating and exposing the immune system to viral proteins, the immune system is often unable to prevent reactivation. CMV and EBV are also frequently shed in saliva (12). Thus, a therapeutic vaccine for these viruses may need to induce immune responses more potent than those occurring with natural immunity.

A common feature of HSV, CMV, and EBV that is not shared with VZV is the large number of immune evasion genes that HSV, CMV, and EBV express that may allow reactivation despite a robust immune response. These viruses encode proteins that inhibit interferon, antibody and complement function, virus antigen presentation to MHC class I and II, and NK cell activity (13). In contrast, VZV is the smallest of the human herpesviruses, and while it has some immune evasion genes, it lacks orthologs for many of the immune evasion genes encoded by the other herpesviruses. Thus, it may be easier for the immune system and a vaccine to prevent reactivation of VZV than other human herpesviruses.

## Selecting targets for therapeutic vaccines for herpesviruses

Using lessons learned from therapy with virus-specific T cells, one might choose the same epitopes used to generate these cells as target epitopes for CMV and EBV therapeutic vaccines. Posoleucel T cells target CMV immediate-early 1 (IE1) and the pp65 tegument proteins, EBV IE BZLF1, and the EBNA1 and LMP2 latency proteins.

**Figure 1:**
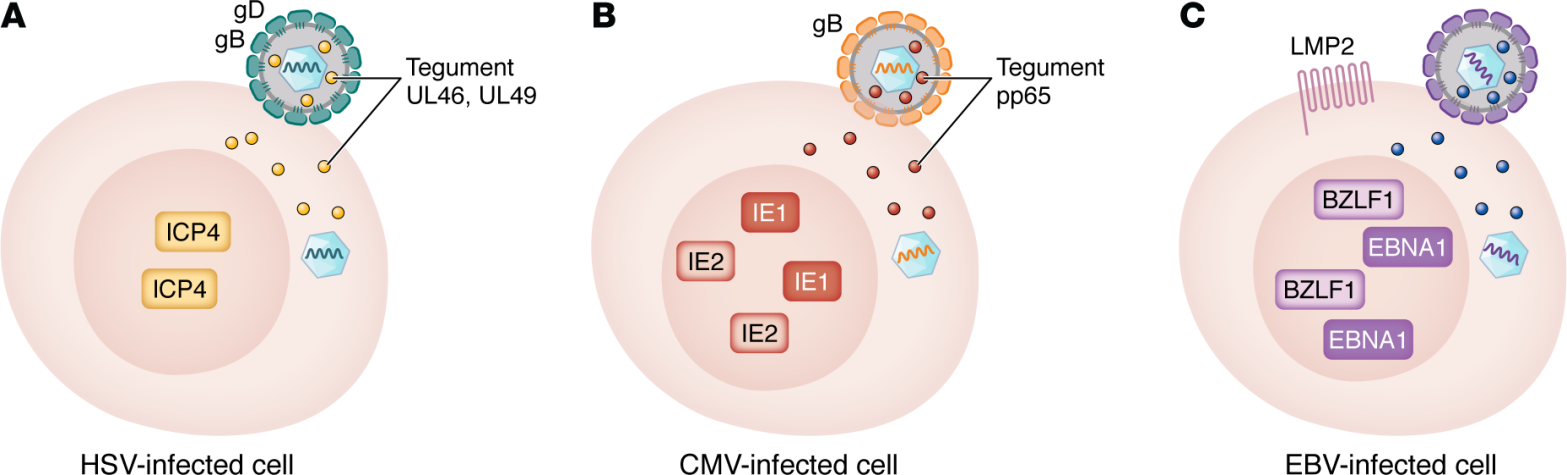
Viral components for a therapeutic vaccine on infected cells. (**A**) Herpes simplex virus– (glycoprotein D [gD], gB, immediate-early [IE] ICP4, UL46, and UL49 tegument proteins), (**B**) CMV- (IE1, IE2, pp65 tegument protein, gB), and (**C**) EBV-infected cells (IE BZLF1 protein, LMP2, EBNA1). Proteins are shown based on their location in infected cells, but viral peptides are presented on the surface of the cells with MHC class I or II.

Several approaches have been used to select targets for therapeutic herpesvirus vaccines (Table 2). The first approach is to identify viral proteins that are frequently recognized as T cell targets in most individuals. An early study showed that the most frequent targets of HSV-2–specific CD8^+^ T cells in peripheral blood were (in descending order) UL39 (large subunit of ribonucleotide reductase), UL25 capsid protein, glycoprotein B (gB), IE protein ICP0, and tegument proteins (UL46 and UL47) (14). HSV glycoproteins (gD, gB), tegument, and IE proteins have been the major focus for HSV therapeutic candidate vaccines (Figure 1A). A similar study for CMV showed that the most frequent targets for virus-specific CD8^+^ T cells were (in descending order) UL48 and pp65 tegument proteins and IE1 and IE2 proteins, while the most common targets for CD4^+^ T cells were (in descending order) gB, pp65, the UL86 capsid protein, the UL99 tegument protein, and IE2 protein (15). pp65, IE1 and IE2 proteins, and gB have been the principal immunogens used in recent therapeutic trials of CMV vaccines (Figure 1B). Studies for EBV showed that the most common targets for CD8 cells are IE proteins BZLF1 and BRLF1, BMRF1 protein (a component of the polymerase), and the EBNA3 latency protein (16). The most common targets for EBV CD4 cells are the EBNA1, EBNA2, and EBNA3 latency proteins. EBNA1 and LMP2 have been the major targets for therapeutic EBV vaccines (Figure 1C).

Another approach is to look at T cell targets in persons who are immune seronegative. These individuals have been exposed to the virus and have T cell responses to viral proteins, but they have not been infected, since they do not have antibody to the virus and do not have detectable virus in the blood or secretions. Immune seronegative persons been described in persons exposed to HIV (17), HSV (18, 19), and EBV (20) but who were not infected. At present it is uncertain if immune seronegative persons are actually protected from infection and, if so, how long such protection lasts. The most common targets for HSV-specific T cells in immune seronegative persons in one study were UL39, followed by IE ICP4 and ICP0 proteins, and UL29 (the major DNA-binding protein) (18).

Additional analyses have been performed for HSV, studying persons who differ in the frequency of symptomatic reactivations; similar analyses have not been performed with CMV and EBV because reactivation is nearly always asymptomatic in healthy persons. A better understanding of why some persons have only asymptomatic HSV reactivations, while others have frequent symptomatic reactivations, may provide important clues for developing HSV therapeutic vaccines. A combination of analyses of T cell responses to HSV-2 proteins included (a) overall frequency of T cell responses in seropositive persons, (b) responses in infected persons who were asymptomatic versus symptomatic, (c) responses that were higher in immune seronegative versus symptomatic persons, and (d) responses that were higher in protected versus unprotected persons (based on their ability to resist infection after known exposure, have asymptomatic infection, or not to have recurrent disease) (19). Using these analyses, the HSV proteins most often targeted by both CD4^+^ and CD8^+^ T cells were ICP4, UL2 (uracil DNA glycosylase), UL11 (a tegument protein), and UL40 (the small subunit of the ribonucleotide kinase). The preponderance of viral proteins that target IE and tegument proteins may reflect their early expression during infection or their presence in virions that infect the cells, resulting in their exposure to T cells before viral immune evasion molecules can block recognition of viral proteins.

Another approach for therapeutic vaccines is to target immune evasion molecules. As noted above, many herpesviruses encode proteins that inhibit immune responses. An HSV mRNA vaccine, which encodes gE and gC, that inhibits antibody and complement activity, respectively, is in a phase I clinical trial (21) (ClinicalTrials.gov NCT05432583).

In addition to virus-specific T cells, antibody effector functions may also be important for killing virus-infected cells (Table 3). ADCC, complement-mediated cellular cytotoxicity, and antibody-dependent cellular phagocytosis may contribute to killing of herpesvirus-infected cells in addition to T cells (22). At present, it is uncertain what the relative contribution of antibody effector functions might be compared with virus-specific T cells in preventing virus reactivation.

## Importance of location of virus-specific T cells in HSV

Virus-specific CD8^+^ T cells should be present at or near the site of reactivation for a vaccine to reduce reactivation of genital or orolabial HSV. HSV-2–specific cells, including CD8 tissue-resident memory T cells, are present in genital mucosa and increase in number during reactivation (23, 24). Nearly all studies of viral proteins important for recognition by T cells are performed using cells from peripheral blood. During reactivation, HSV-2–specific memory T cells enter tissues from the circulation and supplement tissue memory T cell responses (25). Cutaneous lymphocyte-associated antigen (CLA) is a homing marker expressed on HSV-specific CD8^+^ T cells (26), and it is likely important for their ability to traffic to the skin. In 2003, Koelle et al. isolated HSV-2–specific CD8^+^ T cell clones that expressed high levels of CLA from 10 patients infected with HSV-2 (26). These clones recognized viral tegument proteins (52% of clones), capsid proteins (17%), IE proteins (13%), glycoproteins (3%), and other proteins (14%).

Viral proteins targeted by T cells may not be the same for those in the blood and at the sites of virus reactivation. In 2001, Koelle et al. studied virus-specific T cell responses from genital lesions of 3 patients and found that the most common responses were to tegument proteins UL47, UL49, and ICP0 (27). A follow-up study of genital tissue from 8 patients found that the most T cell common responses were to gD (6 patients), gB and UL39 (4 patients), and UL23 (the viral thymidine kinase, 3 patients) (28).

Strategies to induce trafficking of virus-specific T cells from the peripheral blood to the genital mucosa have been successfully employed in animal models. This involves (a) priming animals with peripheral vaccination and (b) pulling T cells to the genital tract by application of chemokines or an immune stimulant. Initial studies in mice used application of chemokines CXCL9 and CXCL10 to selectively recruit CD8 cells to the genital mucosa (29). A study in guinea pigs used topical imiquimod, which recruited both CD8 and CD4 cells (30); increased numbers of CD4 cells in the genital mucosa could potentially increase the risk of infection with HIV.

An additional approach may be the use of a checkpoint inhibitor to enhance a therapeutic vaccine. Addition of an anti–PD-1 antibody to a therapeutic human papillomavirus vaccine enhanced the benefit of the vaccine in an animal model (31), and promising results have been reported in a human trial (32). However, anti–PD-1 antibodies can induce serious immune-mediated complications including colitis, pneumonitis, hepatitis, and endocrinopathies; thus, the risks associated with anti–PD-1 antibodies in otherwise healthy persons receiving a therapeutic vaccine may outweigh their benefits. Most effector CD8^+^ T cells in sensory ganglia express high levels of PD-1 during HSV latency, which correlate with a phenotype of functional exhaustion and increased reactivation ex vivo (33).

## Therapeutic vaccines for HSV

HSV can reactivate in orolabial and genital sites resulting in lesions. In 2023, the NIH issued a document entitled “2023-2028 Strategic Plan for Herpes Simplex Virus Research” which stated that a “A therapeutic vaccine would be intended for patients with an existing HSV infection to elicit an immune response to reduce the frequency of viral reactivation and lesion outbreaks and to reduce viral shedding (34). A therapeutic genital herpes virus vaccine must also reduce shedding; if a vaccine resulted in reduced symptomatic reactivation, but not asymptomatic shedding, it might result in an increase in transmission of virus. While suppressive antiviral therapy with currently licensed drugs reduces reactivation of genital HSV and reduces transmission in otherwise healthy persons by approximately 50%, they must be taken daily to be effective.

Most prophylactic vaccines for HSV have focused on viral glycoproteins gD, gB, gE, and gC (35, 36). Placebo-controlled trials of therapeutic vaccines for HSV-2 using inactivated HSV (37), viral antigens extracted from infected cells (38), HSV glycoproteins extracted from infected cells (39), recombinant gD in alum (40), recombinant gD and gB in MF-59 (41), live-attenuated HSV deleted for UL39 (42), and replication-defective HSV deleted for gH (43) were not successful enough to proceed to further clinical trials. More recently a DNA vaccine expressing both truncated gD that was ubiquitinated and full-length gD (44) resulted in about 50% reduction in viral shedding after booster immunization compared with about 35% for placebo recipients but did not reduce the mean number of outbreaks compared with placebo. A randomized, double-blind, placebo-controlled study of a DNA vaccine expressing gD and UL46 tegument protein reduced new genital lesions by 57% but did not reduce shedding (ClinicalTrials.gov NCT02837575). A vaccine containing 32 HSV-2 peptides and heat shock proteins adjuvanted with saponin induced virus-specific T cell responses in more than 50% of vaccinees and reduced virus shedding by 15% (Agenus; ClinicalTrials.gov NCT01687595) (45).

One of the most promising vaccines was produced by Genocea and contained a portion of ICP4 combined with gD (deleted for its transmembrane domain) adjuvanted in Matrix-M. The vaccine was tested in several clinical trials and induced both neutralizing antibody and polyfunctional virus-specific T cell responses. In the last study performed, when the vaccine was given with two different doses of Matrix-M, it reduced lesion rates by 37%–51% compared with placebo and reduced duration of recurrences by 1.2 days (46). The vaccine significantly reduced virus shedding at 1 year compared with the level of shedding prior to vaccination. The authors concluded that “It remains to be determined if a vaccine strategy that reduces recurrent disease and recurrent virus shedding by about 50%, for about one year, is a viable alternative to antivirals that are more effective but must be taken daily.” The vaccine has not been studied further.

At present, two therapeutic vaccine trials are ongoing. One is a dose-ranging study of an HSV mRNA (Moderna, ClinicalTrials.gov NCT06033261), and another is a vaccine whose composition has not been disclosed (GSK, ClinicalTrials.gov NCT05298254).

## Therapeutic vaccines for CMV

CMV is the most common infectious cause of birth defects and the most important viral infection in organ transplant and hematopoietic stem cell transplant (HSCT) recipients. While most prophylactic CMV vaccines target gB and the pentamer complex (gH/gL/UL128-131), most therapeutic vaccines target pp65, IE1 or IE2 proteins, or gB. In contrast to therapeutic vaccines for HSV and VZV, which are targeted primarily to healthy or mildly immunocompromised individuals, therapeutic vaccines for CMV are focused on immunocompromised persons such as transplant recipients. Such persons generally respond less well to vaccines, which may make a successful therapeutic vaccine for CMV particularly difficult to develop.

Suppressive antiviral therapy is used to reduce reactivation and disease associated with CMV in transplant recipients. Currently available antivirals for CMV have concerns, including numerous interactions with other medications (letermovir), bone marrow toxicity (ganciclovir), or renal toxicity (foscarnet). Thus, an effective CMV therapeutic vaccine could avoid the use of medications with serious side effects and concerns about drug interactions.

One of the first therapeutic vaccines in clinical trials was the attenuated Towne strain of CMV. Vaccination of seropositive renal transplant recipients resulted in no benefit; however, seronegative recipients had less severe CMV disease (47). More recent trials have used CMV subunit, DNA, peptide, and viral vector vaccines. Vaccination of seropositive and seronegative kidney or liver transplant recipients with CMV gB in MF59 adjuvant resulted in no protection in CMV-seropositive organ transplant recipients; however, seronegative recipients of organs from seropositive donors had a shorter duration of viremia and required fewer days of antiviral therapy (48). A phase III trial of a DNA vaccine expressing gB and pp65 in CMV-seropositive HSCT transplant recipients showed no effect on CMV end organ disease and all-cause mortality (the primary endpoint), rate of viremia, or use of antiviral therapy (49). A lymphocytic choriomeningitis virus replication-defective vaccine expressing gB and pp65 (ClinicalTrials.gov NCT02798692) was ineffective in CMV-seronegative renal transplant recipients receiving kidneys from CMV-seropositive donors. A poxvirus (canarypox) vector expressing CMV pp65 was tested in HSCT donors in a phase II trial (ClinicalTrials.gov NCT00353977), but the results have not been reported.

A poxvirus (modified vaccinia Ankara) vector expressing CMV pp65, IE1, and IE2 induced virus-specific T cells and reduced cumulative events (virus reactivation, viremia requiring treatment, or CMV organ disease) in CMV-seropositive HSCT recipients by approximately 50% in a phase II trial, although the difference between the vaccine and placebo was not significant (50). This vaccine is currently in several additional phase I/II or phase II trials: in CMV-seropositive children receiving HSCT (ClinicalTrials.gov NCT03354728), in CMV-seropositive adults undergoing HSCT (ClinicalTrials.gov NCT04060277), in HSCT donors (ClinicalTrials.gov NCT06059391), in CMV-seronegative liver transplant recipients (ClinicalTrials.gov NCT06075745), and in HIV- and CMV-seropositive adults (ClinicalTrials.gov NCT05099965).

A vaccine consisting of a CMV pp65 peptide conjugated to a portion of tetanus toxoid with CpG oligonucleotide adjuvant induced virus-specific T cells and significantly reduced CMV viremia, reduced the duration of preemptive antiviral therapy, and increased the duration of relapse-free survival in a phase Ib trial in HLA-A0201–positive CMV-seropositive HSCT recipients compared with patients who were only observed (51). This vaccine is currently in a phase II trial in CMV-seropositive HSCT recipients (ClinicalTrials.gov NCT02396134).

Two CMV mRNA vaccine trials are planned for CMV-seropositive HSCT recipients (ClinicalTrials.gov NCT05683457) and CMV-seropositive or -seronegative liver transplant recipients (ClinicalTrials.gov NCT06133010); these vaccines contain 6 mRNAs that encode gB and the pentamer (gH/gL/UL128-131) complex.

## Therapeutic vaccines for EBV

EBV is associated with several malignancies, including nasopharyngeal carcinoma, gastric carcinoma, Hodgkin lymphoma, Burkitt lymphoma, non–Hodgkin lymphoma, and posttransplant lymphoma. Prophylactic vaccines have focused on gp350 and recently gH/gL/gp42. Therapeutic vaccines have been tested for treatment of EBV malignancies, especially nasopharyngeal carcinoma. Different EBV-associated cancers express different groups of viral proteins; most EBV-positive nasopharyngeal carcinomas express EBV EBNA1, LMP1, and LMP2.

A poxvirus (modified vaccinia Ankara) vector expressing the carboxy portion of EBV EBNA1 and an inactivated form of EBV LMP2 was developed to treat patients with nasopharyngeal carcinoma. This vaccine induced CD4^+^ and CD8^+^ T cell responses to EBNA1 and LMP2, respectively, in patients in the United Kingdom and in Hong Kong with nasopharyngeal carcinoma (52, 53). A phase II trial of the vaccine was completed in patients with nasopharyngeal carcinoma but results have not been posted (ClinicalTrials.gov NCT01094405). A phase I trial of the vaccine for various EBV malignancies (gastric carcinoma, lymphoma, head and neck cancer, lymphoproliferative disease) has also been completed, though no results have been reported (ClinicalTrials.gov NCT01147991).

Another therapeutic EBV vaccine, an adenovirus vector expressing LMP2, was tested in a phase I trial of patients with nasopharyngeal carcinoma in China (54); results of its effect on virus-specific T cells have not been reported.

Peptide vaccines, which contain one of two different LMP2 peptides in Montanide ISA-51 adjuvant were tested in a phase I trial in patients with nasopharyngeal carcinoma that are either HLA-A*1101 or HLA-A*2402; results have not been reported (ClinicalTrials.gov NCT00078494). A vaccine using EBV mRNA for patients with refractory EBV-positive malignancies has been initiated in a phase I trial in China (ClinicalTrials.gov NCT05714748).

## Future directions

Eighteen years after the first therapeutic vaccine for a herpesvirus, the live-attenuated zoster vaccine, was licensed, and six years after the zoster subunit was licensed, there are no other approved therapeutic vaccines for other herpesviruses. Along with morbidity associated with virus reactivation in both healthy and immunocompromised patients, persistent infection with some herpesviruses has been associated with malignancies. The increasing evidence that herpesviruses may be an important cause of chronic inflammation and their association with autoimmune diseases and dementia underscores the importance of developing vaccines. Numerous advances in vaccine development, including viral vectored vaccines, mRNA vaccines, adjuvants that induce CD4^+^ and CD8^+^ T cell responses, structural biology to identify more immunogenic conformations of viral proteins, and new approaches for mucosal vaccines provide optimism that additional therapeutic vaccines for herpesviruses will be successful.

## Figures and Tables

**Table 3:**
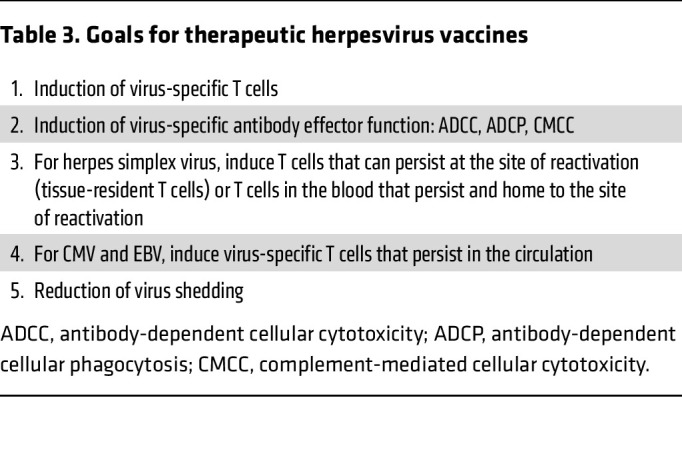
Goals for therapeutic herpesvirus vaccines

**Table 2:**
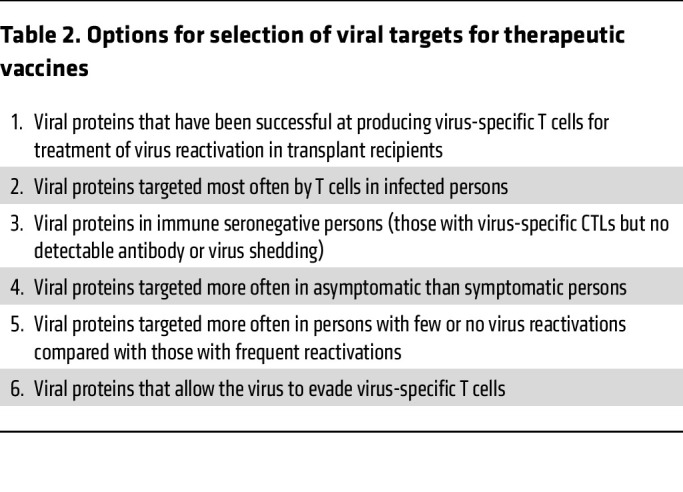
Options for selection of viral targets for therapeutic vaccines

**Table 1:**
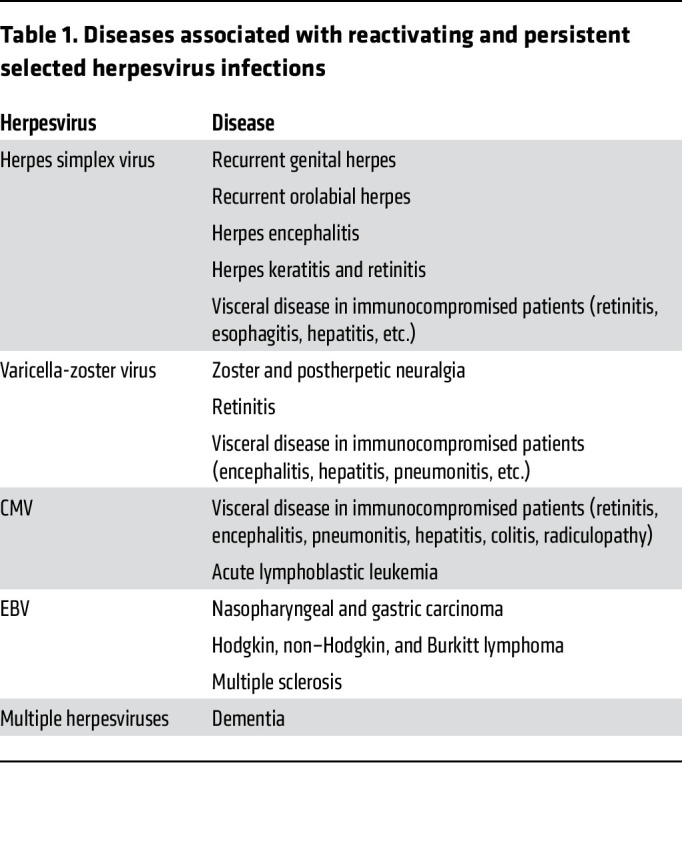
Diseases associated with reactivating and persistent selected herpesvirus infections

## Version 1 05/01/2024

Electronic publication

## References

[1] Bjornevik K (2022). Longitudinal analysis reveals high prevalence of Epstein-Barr virus associated with multiple sclerosis. Science.

[2] Warren-Gash C (2019). Human herpesvirus infections and dementia or mild cognitive impairment: a systematic review and meta-analysis. Sci Rep.

[4] Plotkin SA (2010). Correlates of protection induced by vaccination. Clin Vaccine Immunol.

[5] Weinberg A (2009). Varicella-zoster virus-specific immune responses to herpes zoster in elderly participants in a trial of a clinically effective zoster vaccine. J Infect Dis.

[6] Harbecke R (2021). Herpes zoster vaccines. J Infect Dis.

[7] Levin MJ (2018). Th1 memory differentiates recombinant from live herpes zoster vaccines. J Clin Invest.

[8] Park SY (2022). Development of antibody-dependent cellular cytotoxicity in response to recombinant and live-attenuated herpes zoster vaccines. NPJ Vaccines.

[9] Pfeiffer T (2023). Posoleucel, an allogeneic, off-the-shelf multivirus-specific T-cell therapy, for the treatment of refractory viral infections in the post-HCT setting. Clin Cancer Res.

[10] Leen AM (2006). Monoculture-derived T lymphocytes specific for multiple viruses expand and produce clinically relevant effects in immunocompromised individuals. Nat Med.

[11] Mark KE (2008). Rapidly cleared episodes of herpes simplex virus reactivation in immunocompetent adults. J Infect Dis.

[12] de França TR (2012). Salivary shedding of Epstein-Barr virus and cytomegalovirus in people infected or not by human immunodeficiency virus 1. Clin Oral Investig.

[13] Tortorella D (2000). Viral subversion of the immune system. Annu Rev Immunol.

[14] Hosken N (2006). Diversity of the CD8^+^ T-cell response to herpes simplex virus type 2 proteins among persons with genital herpes. J Virol.

[15] Sylwester AW (2005). Broadly targeted human cytomegalovirus-specific CD4^+^ and CD8^+^ T cells dominate the memory compartments of exposed subjects. J Exp Med.

[16] Taylor GS (2015). The immunology of Epstein-Barr virus-induced disease. Annu Rev Immunol.

[17] Kaul R (2000). HIV-1-specific mucosal CD8^+^ lymphocyte responses in the cervix of HIV-1-resistant prostitutes in Nairobi. J Immunol.

[18] Posavad CM (2010). Detailed characterization of T cell responses to herpes simplex virus-2 in immune seronegative persons. J Immunol.

[19] Long D (2014). Identification of novel virus-specific antigens by CD4^+^ and CD8^+^ T cells from asymptomatic HSV-2 seropositive and seronegative donors. Virology.

[20] Savoldo B (2002). Generation of EBV-specific CD4^+^ cytotoxic T cells from virus naive individuals. J Immunol.

[21] Awasthi S (2019). Nucleoside-modified mRNA encoding HSV-2 glycoproteins C, D, and E prevents clinical and subclinical genital herpes. Sci Immunol.

[22] Jenks JA (2019). The roles of host and viral antibody Fc Receptors in herpes simplex virus (HSV) and human cytomegalovirus (HCMV) infections and immunity. Front Immunol.

[23] Zhu J (2007). Virus-specific CD8^+^ T cells accumulate near sensory nerve endings in genital skin during subclinical HSV-2 reactivation. J Exp Med.

[24] Peng T (2021). Tissue-resident-memory CD8^+^ T cells bridge innate immune responses in neighboring epithelial cells to control human genital herpes. Front Immunol.

[25] Davé V (2023). Recurrent infection transiently expands human tissue T cells while maintaining long-term homeostasis. J Exp Med.

[26] Koelle DM (2003). Immunodominance among herpes simplex virus-specific CD8 T cells expressing a tissue-specific homing receptor. Proc Natl Acad Sci U S A.

[27] Koelle DM (2001). CD8 CTL from genital herpes simplex lesions: recognition of viral tegument and immediate early proteins and lysis of infected cutaneous cells. J Immunol.

[28] Koelle DM (2022). HSV-2-specific human female reproductive tract tissue resident memory T cells recognize diverse HSV antigens. Front Immunol.

[29] Shin H, Iwasaki A (2012). A vaccine strategy that protects against genital herpes by establishing local memory T cells. Nature.

[30] Bernstein DI (2019). Successful application of prime and pull strategy for a therapeutic HSV vaccine. NPJ Vaccines.

[31] Peng S (2021). Development of DNA vaccine targeting E6 and E7 proteins of human papillomavirus 16 (HPV16) and HPV18 for immunotherapy in combination with recombinant vaccinia boost and PD-1 antibody. mBio.

[32] Massarelli E (2019). Combining immune checkpoint blockade and tumor-specific vaccine for patients with incurable human papillomavirus 16-related cancer: a phase 2 clinical trial. JAMA Oncol.

[33] Chentoufi AA (2022). Combinatorial herpes simplex vaccine strategies: from bedside to bench and back. Front Immunol.

[34] https://www.niaid.nih.gov/sites/default/files/nih-herpes-simplex-strategic-plan-2023.pdf.

[35] Dropulic LK, Cohen JI (2012). The challenge of developing a herpes simplex virus 2 vaccine. Expert Rev Vaccines.

[36] Egan K (2020). Vaccines to prevent genital herpes. Transl Res.

[37] Kern AB, Schiff BL (1964). Vaccine therapy in recurrent herpes simplex. Arch Dermatol.

[38] Kutinová L (1988). Placebo-controlled study with subunit herpes simplex virus vaccine in subjects suffering from frequent herpetic recurrences. Vaccine.

[39] Skinner GR (1997). The efficacy and safety of Skinner herpes simplex vaccine towards modulation of herpes genitalis; report of a prospective double-blind placebo-controlled trial. Med Microbiol Immunol.

[40] Straus SE (1994). Placebo-controlled trial of vaccination with recombinant glycoprotein D of herpes simplex virus type 2 for immunotherapy of genital herpes. Lancet.

[41] Straus SE (1997). Immunotherapy of recurrent genital herpes with recombinant herpes simplex virus type 2 glycoproteins D and B: results of a placebo-controlled vaccine trial. J Infect Dis.

[42] Casanova G (2002). A double-blind study of the efficacy and safety of the ICP10δPK vaccine against recurrent genital HSV-2 infections. Cutis.

[43] de Bruyn G (2006). A randomized controlled trial of a replication defective (gH deletion) herpes simplex virus vaccine for the treatment of recurrent genital herpes among immunocompetent subjects. Vaccine.

[44] Chandra J (2019). Immune responses to a HSV-2 polynucleotide immunotherapy COR-1 in HSV-2 positive subjects: A randomized double blinded phase I/IIa trial. PLoS One.

[45] Wald A (2011). Safety and immunogenicity of long HSV-2 peptides complexed with rhHsc70 in HSV-2 seropositive persons. Vaccine.

[46] Bernstein DI (2019). Therapeutic HSV-2 vaccine decreases recurrent virus shedding and recurrent genital herpes disease. Vaccine.

[47] Plotkin SA (1991). Effect of Towne live virus vaccine on cytomegalovirus disease after renal transplant. A controlled trial. Ann Intern Med.

[48] Griffiths PD (2011). Cytomegalovirus glycoprotein-B vaccine with MF59 adjuvant in transplant recipients: a phase 2 randomised placebo-controlled trial. Lancet.

[49] Ljungman P (2021). A randomised, placebo-controlled phase 3 study to evaluate the efficacy and safety of ASP0113, a DNA-based CMV vaccine, in seropositive allogeneic haematopoietic cell transplant recipients. EClinicalMedicine.

[50] Aldoss I (2020). Poxvirus vectored cytomegalovirus vaccine to prevent cytomegalovirus viremia in transplant recipients: a phase 2, randomized clinical trial. Ann Intern Med.

[51] Nakamura R (2016). Viraemia, immunogenicity, and survival outcomes of cytomegalovirus chimeric epitope vaccine supplemented with PF03512676 (CMVPepVax) in allogeneic haemopoietic stem-cell transplantation: randomised phase 1b trial. Lancet Haematol.

[52] Hui EP (2013). Phase I trial of recombinant modified vaccinia ankara encoding Epstein-Barr viral tumor antigens in nasopharyngeal carcinoma patients. Cancer Res.

[53] Taylor GS (2014). A recombinant modified vaccinia ankara vaccine encoding Epstein-Barr Virus (EBV) target antigens: a phase I trial in UK patients with EBV-positive cancer. Clin Cancer Res.

[54] Si Y (2016). The Safety and immunological effects of rAd5-EBV-LMP2 vaccine in nasopharyngeal carcinoma patients: a phase I clinical trial and two-year follow-up. Chem Pharm Bull (Tokyo).

